# Marinomonas rhodophyticola sp. nov. and Marinomonas phaeophyticola sp. nov., isolated from marine algae

**DOI:** 10.1099/ijsem.0.006366

**Published:** 2024-05-03

**Authors:** Mahrukh Butt, Dae Gyu Choi, Jeong Min Kim, Jae Kyeong Lee, Ju Hye Baek, Che Ok Jeon

**Affiliations:** 1 Department of Life Science, Chung-Ang University, Seoul 06974, Republic of Korea

**Keywords:** marine algae, *Marinomonas phaeophyticola*, *Marinomonas rhodophyticola*, novel taxa, *Pseudomonadota*

## Abstract

Two Gram-stain-negative, facultatively aerobic, and motile rod bacteria, designated as strains KJ51-3^T^ and 15G1-11^T^, were isolated from marine algae collected in the Republic of Korea. Both strains exhibited catalase- and oxidase-positive activities. Optimum growth conditions for strain KJ51-3^T^ were observed at 30 °C and pH 6.0–8.0, with 1.0–7.0 % (w/v) NaCl, whereas strain 15G1-11^T^ exhibited optimal growth at 30 °C, pH 7.0, and 1.0–5.0 % NaCl. Major fatty acids detected in both strains included C_16 : 0_, C_10 : 0_ 3-OH and summed features 3 (C_16 : 1_* ω*7*c* and/or C_16 : 1_* ω*6*c*) and 8 (C_18 : 1_* ω*7*c* and/or C_18 : 1_* ω*6*c*). As for polar lipids, strain KJ51-3^T^ contained phosphatidylethanolamine (PE), phosphatidylglycerol (PG), diphosphatidylglycerol, and two unidentified phospholipids, whereas strain 15G1-11^T^ had PE, PG, and an unidentified aminolipid. Ubiquinone-8 was the predominant respiratory quinone in both strains, with minor detection of ubiquinone-9 in strain KJ51-3^T^. The genomic DNA G+C contents were 44.0 mol% for strain KJ51-3^T^ and 40.5 mol% for strain 15G1-11^T^. Phylogenetic analyses based on both 16S rRNA gene and genome sequences placed strains KJ51-3^T^ and 15G1-11^T^ into distinct lineages within the genus *Marinomonas*, most closely related to *Marinomonas arctica* 328^T^ (98.6 %) and *Marinomonas algicola* SM1966^T^ (98.3 %), respectively. Strains KJ51-3^T^ and 15G1-11^T^ exhibited a 94.6 % 16S rRNA gene sequence similarity and a 70.7 % average nucleotide identity (ANI), with ANI values of 91.9 and 79.3 % between them and *M. arctica* 328^T^ and *M. algicola* SM1966^T^, respectively, indicating that they represent novel species. In summary, based on their phenotypic, chemotaxonomic, and phylogenetic properties, strains KJ51-3^T^ and 15G1-11^T^ are proposed to represent novel species within the genus *Marinomonas*, for which the names *Marinomonas rhodophyticola* sp. nov. (KJ51-3^T^=KACC 22756^T^=JCM 35591^T^) and *Marinomonas phaeophyticola* sp. nov. (15G1-11^T^=KACC 22593^T^=JCM 35412^T^) are respectively proposed.

## Introduction

The genus *Marinomonas*, classified within the family *Oceanospirillaceae* of the phylum *Pseudomonadota*, was initially proposed by Van Landschoot and De Ley [1] through the reclassification of two species of the genus *Alteromonas*, *Alteromonas vaga* and *Alteromonas communis*, as *Marinomonas vaga* and *Marinomonas communis* [2]. As of February 2024, the genus *Marinomonas* encompasses 41 validly and two invalidly published species (https://lpsn.dsmz.de/genus/marinomonas), predominantly isolated from marine environments [3,10]. The members of the genus *Marinomonas* are typically Gram-negative, exhibit positive reactions for oxidase and catalase, and feature polar flagellated straight or curved motile rods with non-pigmented to pale pink coloration [1,7]. Additionally, these bacteria usually require Na^+^ for growth and possess C_16 : 0_, C_10 : 0_ 3-OH, summed feature 3 (comprising C_16 : 1_* ω*7*c* and/or C_16 : 1_* ω*6*c*), and summed feature 8 (comprising C_18 : 1_* ω*7*c* and/or C_18 : 1_* ω*6*c*) as their major cellular fatty acids and ubiquinone-8 (Q-8) as their major respiratory quinone [3,10]. In our previous studies on the interactions between marine algae and bacteria, we isolated numerous novel bacteria from marine algae [11,14]. In this study, we isolated and taxonomically characterized two putative novel bacterial strains, designated as strains KJ51-3^T^ and 15G1-11^T^, belonging to the genus *Marinomonas*, using a polyphasic approach.

## Strain isolation

As previously described [11], strains KJ51-3^T^ and 15G1-11^T^ were isolated in June 2021 from the marine red alga *Ahnfeltiopsis flabelliformis* and the marine brown alga *Sargassum thunbergii*, respectively, which were collected from Yangyang-gun (37° 57′ 51.4″ N 128° 45′ 57.6″ E) and Goseong-gun (38° 22′ 22.5″ N 128° 30′ 34.8″ E), respectively, both located in Gangwon Province, Republic of Korea. Briefly, the collected marine algae were gently washed via mechanical vortexing with artificial seawater (ASW; 20 g NaCl, 2.9 g MgSO_4_, 4.5 g MgCl_2_·6H_2_O, 0.6 g KCl, and 1.8 g CaCl_2_·2H_2_O per litre), followed by mechanical homogenization using an Ultra-Turrax homogenizer (IKA) for 10 s. The resulting homogenates were serially diluted in ASW, after which 100 µl aliquots from each dilution were spread on marine agar (MA; MBcell). The agar plates were aerobically incubated at 25 °C for 5 days, and the 16S rRNA genes of colonies grown on MA were amplified via PCR using the 27F (5′-AGAGTTTGATCMTGGCTCAG-3′) and 1492R (5′-TACGGYTACCTTGTTACGACTT-3′) universal primers [11]. PCR amplicons were double-digested with *Hae*III and *Hha*I, followed by analysis via 2 % agarose gel electrophoresis. PCR products displaying distinct fragment patterns were partially sequenced using the 340F (5′-CCTACGGGAGGCAGCAG-3′) universal primer [11], after which the sequences were compared with those of all type strains of validly published species on the EzBioCloud server (www.ezbiocloud.net/identify) [15]. Based on the results from this analysis, two potential novel strains, designated as KJ51-3^T^ and 15G1-11^T^, were selected for further taxonomic characterizations. The strains were routinely cultured on MA for 2 days at 30 °C and preserved at −80 °C in marine broth (MB; MBcell) supplemented with 15 % (v/v) glycerol for long-term preservation. Reference strains *Marinomonas arctica* JCM 14976^T^, *Marinomonas algicola* KCTC 72848^T^, and *Marinomonas communis* DSM 5604^T^ obtained from their respective culture collection centres were used to conduct comparative analyses of genomic characteristics, phenotypic properties, and fatty acid compositions.

## Phylogeny based on 16S rRNA gene sequences

The 16S rRNA gene amplicons from strains KJ51-3^T^ and 15G1-11^T^, amplified using the 27F/1492R primer pair, were subsequently sequenced using the 518R (5′-ATTACCGCGGCTGCTGG-3′) and 805F (5′-GATTAGATACCCTGGTAGTC-3′) primers [11]. By assembling the sequences obtained from the 340F, 518R, and 805F primers, nearly complete 16S rRNA gene sequences were acquired for strains KJ51-3^T^ (1478 nucleotides) and 15G1-11^T^ (1485 nucleotides). Afterward, the similarities between the 16S rRNA gene sequences of strains KJ51-3^T^ and 15G1-11^T^, as well as their closely related type strains, were calculated using EzTaxon (www.ezbiocloud.net/identify) [15]. Alignment of the 16S rRNA gene sequences from strains KJ51-3^T^ and 15G1-11^T^ was performed using the fast secondary-structure-aware infernal aligner (version 1.1.4) [16]. Phylogenetic trees with bootstrap values derived from 1000 replications were reconstructed using the neighbour-joining (NJ), maximum-likelihood (ML), and maximum-parsimony (MP) algorithms in the mega 11 software [17].

**Fig. 1.**
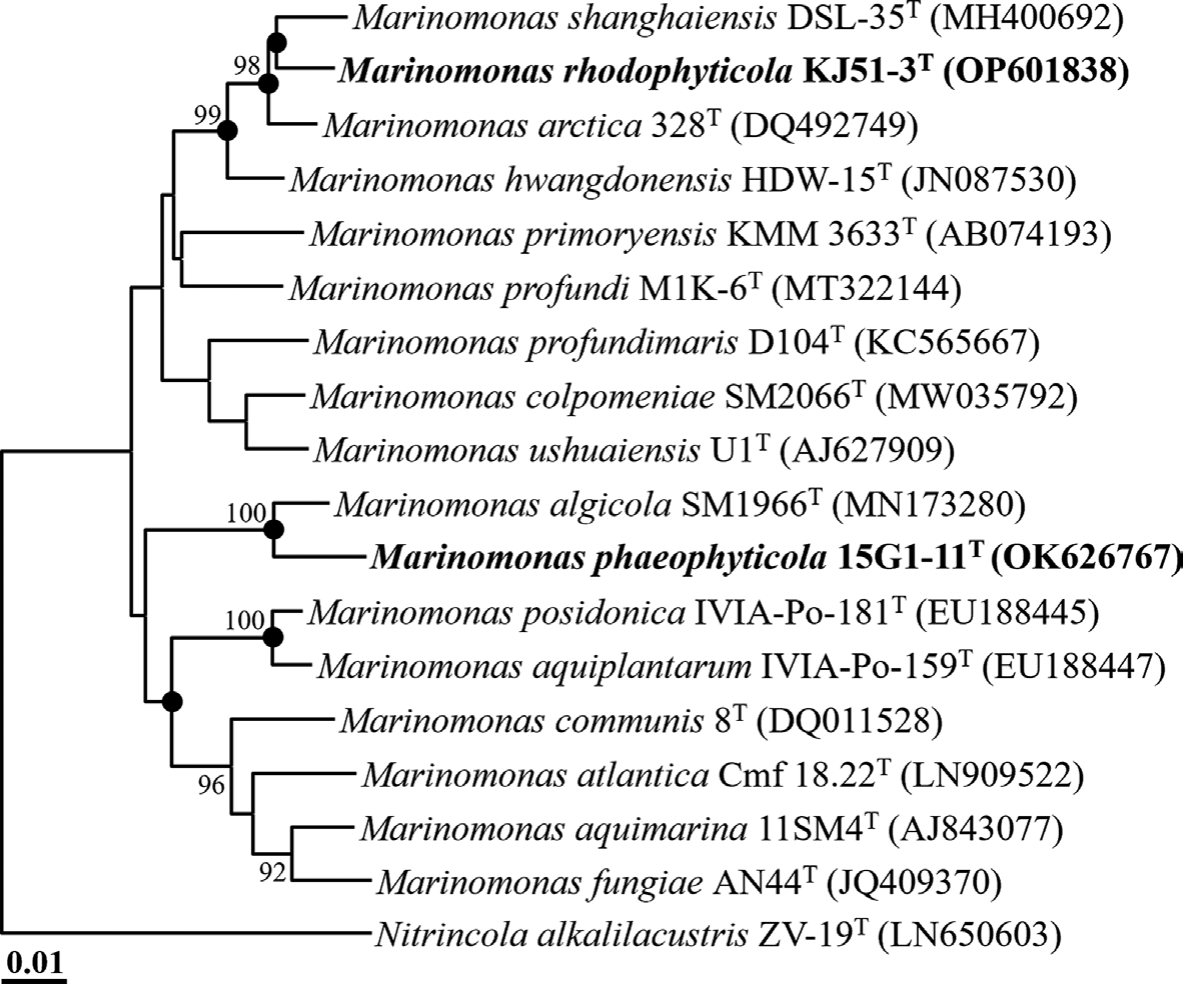
Neighbour-joining tree showing the phylogenetic relationships between strains KJ51-3^T^ and 15G1-11^T^ and their closely related taxa, based on 16S rRNA gene sequences. Filled circles (●) indicate nodes that were also recovered in the maximum-likelihood and maximum-parsimony trees. Only bootstrap values exceeding 70 % are indicated on the nodes as percentages from 1000 replicates. *Nitrincola alkalilacustris* ZV-19^T^ (LN650603) was employed as an outgroup. Bar, 0.01 substitutions per nucleotide.

The 16S rRNA gene sequence comparison between strains KJ51-3^T^ and 15G1-11^T^ yielded a similarity of 94.6 %, which falls below the designated species differentiation threshold of 98.7 % [18]. This suggests that these strains likely represent distinct species. Comparative analysis of their 16S rRNA gene sequences indicated the highest similarities of 98.6 and 98.3 % to *M. arctica* 328^T^ and *M. algicola* SM1966^T^, respectively, suggesting that these strains should be classified as representing novel species. Phylogenetic analysis based on 16S rRNA gene sequences, utilizing the NJ algorithm, revealed that strains KJ51-3^T^ and 15G1-11^T^ formed phyletic lineages with *M. shanghaiensis* DSL-35^T^ and *M. algicola* SM1966^T^, respectively, within the genus *Marinomonas* (Fig. 1). Furthermore, phylogenetic trees generated using ML and MP algorithms further confirmed that strains KJ51-3^T^ and 15G1-11^T^ clustered with members of the genera *Marinomonas* (Fig. S1, available in the online version of this article). These combined results from comparative and phylogenetic analyses based on 16S rRNA gene sequences suggest that strains KJ51-3^T^ and 15G1-11^T^ may be novel members of the genus *Marinomonas*.

## Whole-genome sequencing and genome-based phylogeny

The genomic DNA of strains KJ51-3^T^ and 15G1-11^T^ was extracted from cells cultured in MB using the Wizard Genomic DNA purification kit from Promega, following the manufacturer’s instructions. The extracted DNA was then sequenced using an Oxford Nanopore MinION sequencer. The resulting sequencing reads were *de novo*-assembled using Flye (version 2.9.1) [19], after which the quality of the assembled genomes was assessed based on their completeness and contamination rates using the CheckM program (version 1.0.4) [20]. A genome-based phylogenomic analysis for strains KJ51-3^T^ and 15G1-11^T^, along with closely related type strains, was conducted using the Genome Taxonomy Database Toolkit (GTDB-Tk) based on a set of 120 ubiquitous single-copy marker genes (bac120 marker set) [21]. An ML phylogenomic tree, supported by bootstrap values derived from 1000 replications, was reconstructed based on the concatenated amino acid sequences of these marker genes using the mega 11 software. Additionally, average nucleotide identity (ANI) and digital DNA–DNA hybridization (dDDH) values were calculated between strains KJ51-3^T^ and 15G1-11^T^ and their closely related type strains using the Orthologous Average Nucleotide Identity Tool software available on the EzBioCloud server (www.ezbiocloud.net/sw/oat) [22] and the Genome-to-Genome Distance Calculator version 2.1 (https://ggdc.dsmz.de/distcalc2.php) [23], respectively.

**Fig. 2.**
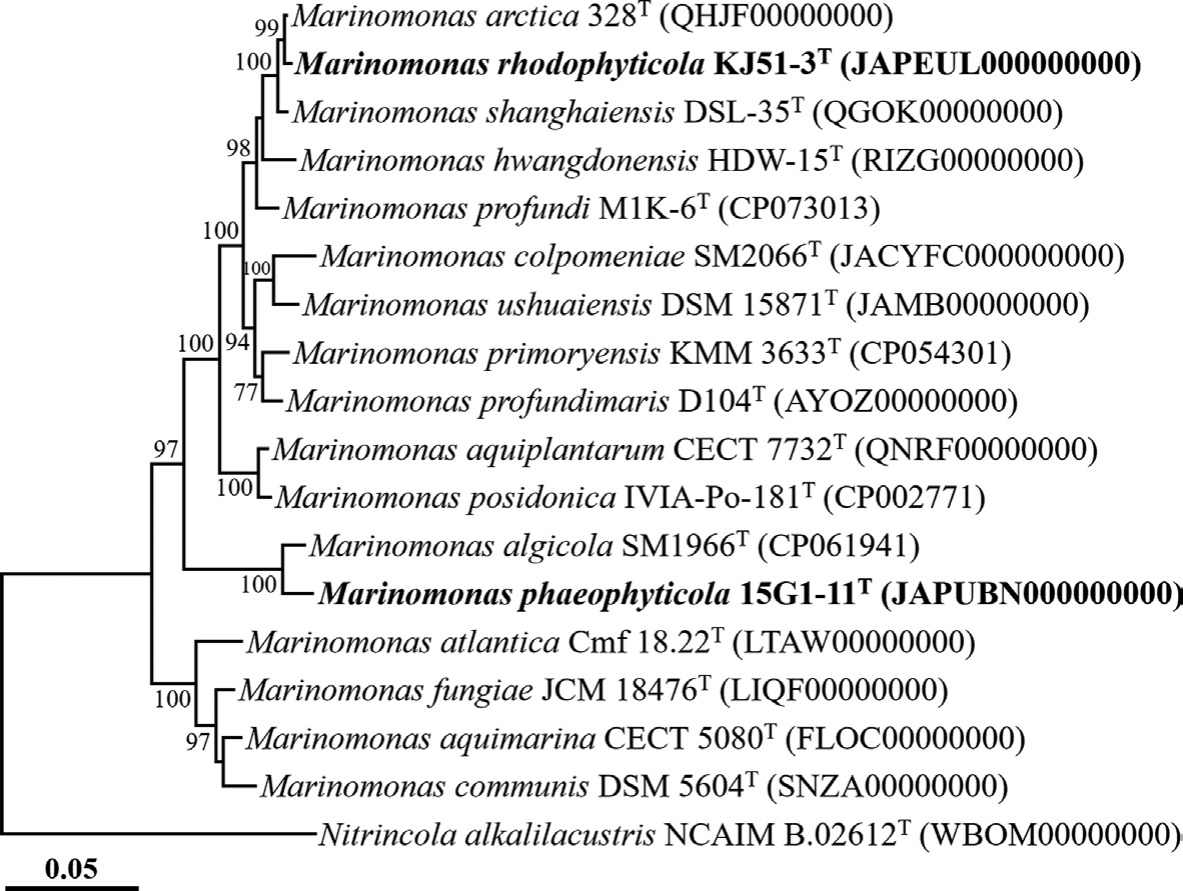
Phylogenomic tree showing the phylogenetic relationships between strains KJ51-3^T^ and 15G1-11^T^ and closely related taxa, based on the concatenation of 120-bacterial protein marker set (bac120 marker set) of GTDB-Tk. Only bootstrap values exceeding 70 % are indicated on the nodes as percentages from 1000 replicates. *Nitrincola alkalilacustris* ZV-19^T^ (WBOM00000000) was employed as an outgroup. Bar, 0.05 substitutions per amino acid.

The *de novo* assembly of the genome sequencing data for strains KJ51-3^T^ and 15G1-11^T^ yielded draft genomes with sizes of 4.6 Mb and 4.1 Mb, comprising 13 contigs and 25 contigs, respectively, and with N50 values of 975.2 kb and 320.0 kb, respectively. The 16S rRNA gene sequences identified in the genomes of both strains were consistent with those obtained through PCR-based sequencing. Assessment of genome completeness and contamination rates for strains KJ51-3^T^ and 15G1-11^T^ revealed values of 99.8 and 98.8 % for completeness, and 5.6 and 0.8 % for contamination rates, respectively, meeting the criteria for generally high-quality genomes (completeness ≥90 % and contamination ≤10 %) [18]. Genome-based phylogenomic analysis demonstrated that strains KJ51-3^T^ and 15G1-11^T^ formed robust phylogenetic lineages with *M. arctica* 328^T^ and *M. algicola* SM1966^T^, respectively, within the genus *Marinomonas*, with bootstrap values of 99 and 100 %, respectively (Fig. 2). The ANI and dDDH values between strains KJ51-3^T^ and 15G1-11^T^ were 70.7 and 21.5 %, respectively, strongly suggesting that these two strains represent distinct species within the genus *Marinomonas*. Moreover, the ANI and dDDH values between strain KJ51-3^T^ and *M. arctica* 328^T^, the closest type strain to strain KJ51-3^T^, were 91.9 and 46.6 %, respectively, whereas the values between strain 15G1-11^T^ and *M. algicola* SM1966^T^, the closest type strain to strain 15G1-11^T^, were 79.3 and 22.7 %, respectively. These values fall substantially below the established thresholds (ANI, ~95 %; dDDH, 70 %) for prokaryotic species delineation [18], indicating that strains KJ51-3^T^ and 15G1-11^T^ represent new species and are distinct from other species members of the genus *Marinomonas*. The findings from the phylogenomic analysis and genome relatedness assessments strongly support the conclusion that strains KJ51-3^T^ and 15G1-11^T^ represent different novel species within the genus *Marinomonas*.

## Genomic features

The genomes of strains KJ51-3^T^ and 15G1-11^T^, along with their reference strains, were annotated using the NCBI Prokaryotic Genome Annotation Pipeline [24]. Subsequently, the carbohydrate-active enzymes (CAZys) were analysed using the dbCAN2 meta server [25]. Strain KJ51-3^T^ was predicted to have a total of 4890 genes, including 4341 protein-coding genes, 128 total RNA genes, 90 tRNA genes, and 27 rRNA genes, whereas strain 15G1-11^T^ was predicted to have a total of 3904 genes, including 3680 protein-coding genes, 119 total RNA genes, 77 tRNA genes, and 20 rRNA genes. The DNA G+C contents of strains KJ51-3^T^ and 15G1-11^T^, calculated from their genomes, were found to be 44.0 and 40.5 mol%, respectively, aligning closely with the G+C contents observed in closely related *Marinomonas* strains (Table 1). Table 1 presents a summary and comparison of the genomic features of these strains with those of closely related type strains of the genus *Marinomonas*, revealing generally similar genomic features among them.

**Table 1.** General genomic features of strains KJ51-3^T^ and 15G1-11^T^ and the type strains of closely related *Marinomonas* species Strains: 1, KJ51-3^T^ (JAPEUL000000000); 2, 15G1-11^T^ (JAPUBN000000000); 3, *M. arctica* 328^T^ (QHJF00000000); 4, *M. algicola* SM1966^T^ (CP061941); 5, *M. communis* DSM 5604^T^ (SNZA00000000). The genomes of strains KJ51-3^T^ and 15G1-11^T^ were sequenced in this study.

Feature*	1	2	3	4	5
Genome status (no. of contigs)^†^	D (13)	D (25)	D (115)	C (2)	D (15)
Genome size (Mb)	4.6	4.1	4.5	4.5	3.9
G+C content (mol%)	44.0	40.5	44	41.5	45.5
N50 (kb)	975.2	320	142.2	4300	461.7
No. of total genes	4890	3904	4201	4119	3610
No. of protein-coding genes	4341	3680	4073	3956	3515
No. of total RNA genes	128	119	92	116	84
No. of tRNA genes	90	77	79	84	69
No. of rRNA genes	27	20	8	28	11
No. of total CAZy^‡^ genes	81	56	73	69	61
Glycoside hydrolase	34	25	34	25	22
Glycosyltransferase	23	18	24	29	25
Polysaccharide lyase	4	1	2	1	1
Carbohydrate esterase	7	6	4	7	6
Auxiliary activities	9	6	7	7	5
Carbohydrate-binding module	4	0	2	0	2

*The genomic features were analysed using the NCBI Prokaryotic Genome Annotation Pipeline (www.ncbi.nlm.nih.gov/genome/annotation_prok/).

†C, complete; D, draft.

‡CAZy, carbohydrate-active enzyme.

Bacteria inhabiting the phycosphere of marine algae are often able to metabolize algal polysaccharides for growth. Given the frequent isolation of *Marinomonas* species from the phycosphere of marine algae [3,7, 8], we conducted a comprehensive genome analysis to investigate the distribution of carbohydrate-active enzyme (CAZy)-encoding genes, potentially involved in the degradation of algal cell-wall constituents, in strains KJ51-3^T^ and 15G1-11^T^, as well as in closely related *Marinomonas* strains. In the genome of strain KJ51-3^T^, 81 putative CAZy-encoding genes were identified, whereas 56 putative CAZy-encoding genes were identified in the genome of strain 15G1-11^T^. These numbers are consistent with those observed in reference strains of the genus *Marinomonas* (Table 1), indicating that strains KJ51-3^T^ and 15G1-11^T^, along with other *Marinomonas* strains, may possess the ability to utilize algal polysaccharides. Further classification of CAZy-encoding genes revealed six distinct classes: glycoside hydrolase (GH; 34 and 25, respectively), glycosyltransferase (GT; 23 and 18, respectively), polysaccharide lyase (PL; four and one, respectively), carbohydrate esterase (CE; seven and six, respectively), auxiliary activities (AA; nine and six, respectively), and carbohydrate-binding module (CBM; four and zero, respectively), with profiles similar to those observed in reference strains of the genus *Marinomonas* (Table 1). Notably, glycoside hydrolases and glycosyltransferases were abundantly identified in all strains among the six major CAZy categories, whereas the carbohydrate-binding module was absent in strain 15G1-11^T^ and the reference strain *M. algicola* SM1966^T^. These findings indicate that strains KJ51-3^T^ and 15G1-11^T^, along with their reference strains, likely share similar capabilities for metabolizing algal polysaccharides.

## Morphology and phenotypic properties

Growth of strains KJ51-3^T^ and 15G1-11^T^ was evaluated on various standard bacteriological agar media (all purchased from MBcell), including MA, Reasoner’s 2A (R2A) agar, Luria–Bertani (LB) agar, tryptic soy agar (TSA), and nutrient agar (NA), all supplemented with 2 % NaCl, and incubated at 30 °C for 2 days. To identify the growth conditions for the isolated strains, their growth rates were assessed at varying temperatures (5–45 °C at 5 °C intervals) and pH values (pH 4.0–11.0 at 1.0 pH unit intervals at 30 °C) on MA and in MB for 2 days, respectively. Media with the desired pH levels were prepared using sodium citrate (pH 4.0–5.0), sodium phosphate (pH 6.0–8.0), and sodium carbonate-bicarbonate (pH 9.0–11.0) buffers, followed by pH readjustment post-sterilization (121 °C for 15 min) if necessary. Salt tolerance was assessed in MB with varying NaCl concentrations (0%–15 % at 1.0 % intervals, w/v) prepared in the laboratory according to the MB composition. Physiological and biochemical tests for strains KJ51-3^T^ and 15G1-11^T^ were conducted using cells cultured for 2 days at 30 °C. Cellular morphology and flagella motility were examined using a phase-contrast microscope (Zeiss Axio Scope. A1, Carl Zeiss). Furthermore, for a detailed examination of cell morphology, size, and flagella, cells cultivated on MA for 2 days were affixed to formvar-coated copper grids, negatively stained with 2 % (w/v) uranyl acetate (Sigma-Aldrich) for 15 s, and subsequently examined under a transmission electron microscope (JEM-1010, jeol). Gram staining was performed using a Gram stain kit from bioMérieux, according to the manufacturer’s instructions. Anaerobic growth of strains KJ51-3^T^ and 15G1-11^T^ was assessed after streaking them on MA plates and incubating them for 21 days at 30 °C under anaerobic conditions created using the GasPak Plus system (BBL). Catalase and oxidase activities were assessed by observing oxygen bubble production in a 3 % (v/v) aqueous hydrogen peroxide solution (Junsei) and the oxidation of 1 % (w/v) tetramethyl-*p*-phenylenediamine (Merck), respectively [26]. The phenotypic characteristics of strains KJ51-3^T^ and 15G1-11^T^ were examined alongside reference strains under the same conditions at their respective optimal temperatures. Hydrolysis of casein (1 % skimmed milk, w/v), starch (1 %), aesculin (0.1 %), Tween 20(1 %), and Tween 80(1 %) was assessed on MA, following previously established protocols [26]. Additional biochemical features and enzymatic activities were evaluated using the API 20NE kit from bioMérieux, according to the manufacturer’s instructions, except that the NaCl concentrations in the solutions provided in the API kit solution were adjusted to approximately 2 % (w/v).

Strains KJ51-3^T^ and 15G1-11^T^ exhibited optimal growth on MA, with favourable growth also observed on R2A agar, TSA, LB agar, and NA supplemented with 2 % NaCl. Morphologically, the cells of strain KJ51-3^T^ appeared as motile rods with a single polar flagellum, measuring 0.5–0.6×1.2–2.0 µm, whereas cells of strain 15G1-11^T^ were also motile rods with a single polar flagellum, measuring 1.0–1.3×2.0–2.7 µm (Fig. S2). The flagellar motility of strains KJ51-3^T^ and 15G1-11^T^ was confirmed through observations under a phase-contrast microscope and motility tests on MA media containing 0.3 % agar. Both strains exhibited minor anaerobic growth, indicating their facultative aerobic nature. Although strains KJ51-3^T^ and 15G1-11^T^ and their closely related reference strains exhibited similarities in several phenotypic, physiological, and biochemical features (e.g. colony colour, oxidase, catalase, and gelatinase activities, nitrate reduction, indole production, d-glucose fermentation, hydrolysis of starch and casein, and assimilation of d-glucose, d-mannose, d-mannitol, maltose, malic acid, and capric acid), they also exhibited discernible differences such as growth conditions and hydrolysis capabilities (e.g. aesculin, Tween 20, and Tween 80), allowing for their distinction from closely related *Marinomonas* type strains (Table 2).

**Table 2.** Differential phenotypic characteristics between strains KJ51-3^T^ and 15G1-11^T^ and the type strains of closely related *Marinomonas* species Strains: 1, KJ51-3^T^; 2, 15G1-11^T^; 3, *M. arctica* 328^T^ [4]; 4, *M. algicola* SM1966^T^ [7]; 5, *M. communis* DSM 5604^T^ [2]. All strains are positive for the following characteristics: flagellum motility, activity* of catalase and oxidase, assimilation* of d-glucose, d-mannose, d-mannitol, maltose, and malic acid. All strains are negative for the following characteristics: nitrate reduction, indole production, d-glucose fermentation, activity* of gelatinase, hydrolysis* of starch and casein, and assimilation* of capric acid. +, Positive; –, negative.

Characteristic	1	2	3	4	5
Isolation source	Red alga	Brown alga	Seawater	Green alga	Seawater
Growth range of:					
Temperature (°C)	15–40	15–35	0–37	5–30	5–30
NaCl (%)	0–13	0–10	0–12	0.5–8.5	0–10
pH	5–9	5–10	5–10	6–10	5–10
Hydrolysis* of:					
Aesculin	+	–	+	–	–
Tween 20	–	+	–	–	–
Tween 80	–	+	–	+	+
Enzyme activity* of:					
β-Galactosidase	+	–	+	–	–
Urease, arginine dihydrolase	+	–	–	+	–
Assimilation* of:					
l-Arabinose	+	–	+	–	–
*N*-Acetyl-glucosamine	–	+	–	+	+
Potassium gluconate	+	–	+	+	–
Adipic acid	+	–	–	–	–
Trisodium citrate	–	+	+	–	–
Phenylacetic acid	–	–	+	–	–

*These data were obtained from this study.

## Chemotaxonomic characteristics

For the analysis of respiratory quinones, strains KJ51-3^T^ and 15G1-11^T^ were cultivated in MB at 30 °C until reaching their exponential growth phases. Microbial cells were then harvested by centrifugation, and respiratory quinones were extracted following the method described by Minnikin *et al.* [27] and analysed using an HPLC system (LC-20A, Shimadzu) equipped with a reversed-phase column (250×4.6 mm, Kromasil, Akzo Nobel) and a diode array detector (SPD-M20A, Shimadzu). Methanol-isopropanol (2 : 1, v/v) served as the eluent at a flow rate of 1 ml min^−1^. For cellular fatty acid analysis, strains KJ51-3^T^ and 15G1-11^T^, alongside their reference strains, were aerobically cultivated in MB under optimal growth temperatures until reaching the exponential growth phase (OD_600_=0.7–0.8). Cellular fatty acids were saponified, methylated, and extracted using the standard procedure outlined in midi (Sherlock Microbial Identification System, version 6.2B). Fatty acid methyl esters were analysed using a gas chromatograph (Hewlett Packard 6890) and identified with the RTSBA6 database of the Microbial Identification System (Sherlock version 6.0B) [28]. Polar lipids were extracted from cells harvested during the exponential growth phase and analysed using two-dimensional thin-layer chromatography, following the procedure by Minnikin *et al.* [29]. Different polar lipids were identified using 10 % ethanolic molybdophosphoric acid (for total polar lipids), ninhydrin (for aminolipids), Dittmer–Lester reagent (for phospholipids), and *α*-naphthol/sulphuric acid (for glycolipids). The presence of phosphatidylethanolamine (PE), phosphatidylglycerol (PG), and diphosphatidylglycerol (DPG) in strains KJ51-3^T^ and 15G1-11^T^ was confirmed using standard polar lipid compounds obtained from Sigma-Aldrich.

Q-8 was identified as the sole respiratory quinone in strain 15G1-11^T^. In strain KJ51-3^T^, Q-8 was predominantly detected, but a minor amount of ubiquinone-9 (Q-9) was also present. The prevalence of Q-8 as the main respiratory quinone in both strains KJ51-3^T^ and 15G1-11^T^ is consistent with its predominance in other members of the genus *Marinomonas* [3,10]. Regarding cellular fatty acids, C_16 : 0_, C_10 : 0_ 3-OH, summed feature 3 (comprising C_16 : 1_* ω*7*c* and/or C_16 : 1_* ω*6*c*), and 8 (comprising C_18 : 1_* ω*7*c* and/or C_18 : 1_* ω*6*c*) were identified as major fatty acids (those constituting >5 % of the total fatty acids) in both strains (Table S2). The overall fatty acid profiles of strains KJ51-3^T^ and 15G1-11^T^, as well as those of reference *Marinomonas* type strains, were generally similar, with only slight variations in the proportions of fatty acids. In strain KJ51-3^T^, PE, PG, and DPG, along with two unidentified phospholipids, were identified as the major polar lipids, whereas the major polar lipids in strain 15G1-11^T^ were PE and PG, along with an unidentified aminolipid (Fig. S3). Overall, the identification of PE and PG as the major polar lipids in strains KJ51-3^T^ and 15G1-11^T^ is consistent with previous observations in other *Marinomonas* species [4,10].

## Taxonomic conclusion

In conclusion, our comprehensive analyses encompassing phylogenetic, genomic relatedness, phenotypic, physiological, biochemical, and chemotaxonomic aspects strongly support the classification of strains KJ51-3^T^ and 15G1-11^T^ as representing novel species within the genus *Marinomonas*. As such, we propose the names *Marinomonas rhodophyticola* sp. nov. and *Marinomonas phaeophyticola* sp. nov., respectively.

## Description of *Marinomonas rhodophyticola* sp. nov.

*Marinomonas rhodophyticola* [rho.do.phy.ti′co.la. N.L. neut. pl. n. *Rhodophyta*, the division of the red algae; L. suffix. -*cola* (from L. masc. or fem. n. *incola*), inhabitant, dweller; N.L. fem. n. *rhodophyticola*, inhabitant of *Rhodophyta*].

Colonies on MA exhibit white, circular, smooth, and shiny morphology. Cells are Gram-stain-negative, facultatively aerobic, rod-shaped, and motile by a single polar flagellum. Growth occurs at 15–40 °C (optimum, 30 °C) and pH 5.0–9.0 (optimum, pH 6.0–8.0) and in the presence of 0.0–13.0 % (w/v) NaCl (optimum, 1.0–7.0 %). Oxidase- and catalase-positive. Nitrate is not reduced. Aesculin is hydrolysed, but starch, casein, Tween 20, and Tween 80 are not Indole production and d-glucose fermentation are negative. Positive for arginine dihydrolase, *β*-galactosidase, and urease activities, but negative for gelatinase activity. Assimilation of d-glucose, l-arabinose, d-mannose, d-mannitol, maltose, potassium gluconate, malic acid, and adipic acid is positive, but assimilation of *N*-acetyl-glucosamine, trisodium citrate, phenylacetic acid, and capric acid is negative. The major respiratory quinone is Q-8, with a minor amount of Q-9. Major fatty acids (>5 %) are C_16 : 0_, C_10 : 0_ 3-OH, summed feature 3 (C_16 : 1_* ω*7*c* and/or C_16 : 1_* ω*6*c*), and summed feature 8 (C_18 : 1_* ω*7*c* and/or C_18 : 1_* ω*6*c*). PE, PG, DPG, and two unidentified phospholipids are detected as major polar lipids.

The type strain is KJ51-3^T^ (=KACC 22756^T^=JCM 35591^T^), isolated from a marine red alga, *Ahnfeltiopsis flabelliformis*, collected in the Republic of Korea. The genome size and DNA G+C content of the type strain are 4.6 Mb and 44.0 mol% (calculated from the whole genome sequence), respectively. The GenBank accession numbers of the 16S rRNA gene and genome sequences of strain KJ51-3^T^ are OP601838 and JAPEUL000000000, respectively.

## Description of *Marinomonas phaeophyticola* sp. nov

*Marinomonas phaeophyticola* [phae.o.phy.ti′co.la. N.L. neut. pl. n. *Phaeophyta,* the division of the brown algae; L. suff. -*cola* (from L. masc. or fem. n. *incola*), inhabitant, dweller; N.L. fem. n. *phaeophyticola*, inhabitant of *Phaeophyta*].

Colonies on MA exhibit smooth, circular, and white morphology. Cells are Gram-stain-negative, facultatively aerobic, rod-shaped, and motile by a single polar flagellum. Growth occurs at 15–35 °C (optimum, 30 °C) and pH 5.0–10.0 (optimum, pH 7.0) and in the presence of 0.0–10.0 % (w/v) NaCl (optimum, 1.0–5.0 %). Oxidase- and catalase-positive. Nitrate is not reduced. Tween 20 and Tween 80 are hydrolysed, but starch, aesculin, and casein are not. Indole production and d-glucose fermentation are negative. Negative for arginine dihydrolase, *β*-galactosidase, urease, and gelatinase activities. Assimilation of d-glucose, d-mannose, d-mannitol, *N*-acetyl-glucosamine, maltose, malic acid, and trisodium citrate is positive, but assimilation of l-arabinose, potassium gluconate, phenylacetic acid, adipic acid, and capric acid is negative. Only Q-8 is detected as the respiratory quinone. Major fatty acids (>5 %) are C_16 : 0_, C_10 : 0_ 3-OH, summed feature 3 (C_16 : 1_* ω*7*c* and/or C_16 : 1_* ω*6*c*), and summed feature 8 (C_18 : 1_* ω*7*c* and/or C_18 : 1_* ω*6*c*). PE, PG, and an unidentified aminolipid are detected as major polar lipids.

The type strain is 15G1-11^T^ (=KACC 22593^T^=JCM 35412^T^), isolated from a brown alga, *Sargassum thunbergii*, collected in the Republic of Korea. The genome size and DNA G+C content of the type strain are 4.1 Mb and 40.5 mol% (calculated from the whole genome sequence), respectively. The GenBank accession numbers of the 16S rRNA gene and genome sequences of strain 15G1-11^T^ are OK626767 and JAPUBN000000000, respectively.

## supplementary material

10.1099/ijsem.0.006366 Uncited Supplementary Material 1.

## Abbreviations

ANI: average nucleotide identity
ASW: artificial seawater
CAZy: carbohydrate-active enzyme
dDDH: digital DNA–DNA hybridization
DPG: diphosphatidylglycerol
MA: marine agar
MB: marine broth
ML: maximum-likelihood
MP: maximum-parsimony
NJ: neighbour-joining
PE: phosphatidylethanolamine
PG: phosphatidylglycerol
Q-8: ubiquinone-8
Q-9: ubiquinone-9
