# Supplementary material for: Marinomonas rhodophyticola sp. nov. and Marinomonas phaeophyticola sp. nov., isolated from marine algae
Source: Int J Syst Evol Microbiol. 2024 May 3;74(5):006366. doi: 10.1099/ijsem.0.006366 (PMC11165874; doi:10.1099/ijsem.0.006366)
Supplement: Uncited Supplementary Material 1. [file ijsem-74-06366-s001.pdf]

Supplementary Information

Fig. S1. Maximum-likelihood (A) and maximum-parsimony (B) trees showing the phylogenetic relationships of strains KJ51-3^T and 15G1-11^T and their closely related taxa, based on 16S rRNA gene sequences. Only bootstrap values exceeding 70% are indicated on the nodes as percentages from 1000 replicates. *Nitrincola alkalilacustris* ZV-19^T (LN650603) was employed as an outgroup. The scale bars in panels A and B represent substitutions per nucleotide and over the entire sequences, respectively.

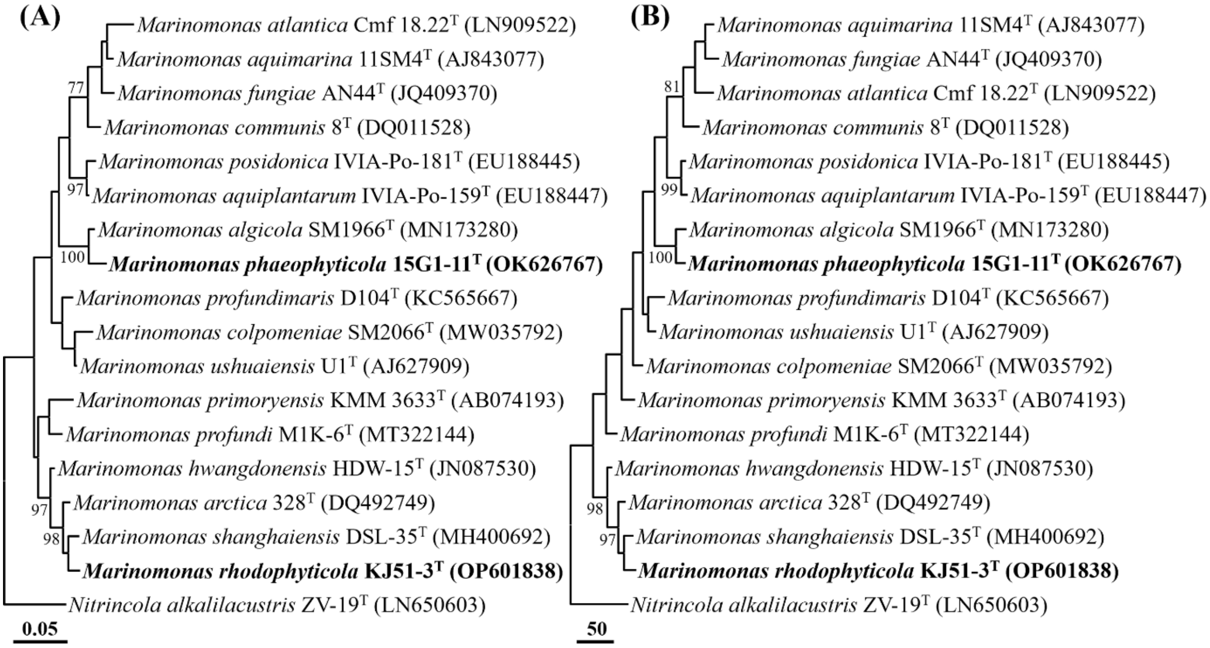

Fig. S2. Transmission electron micrographs showing the general cellular morphology of negatively stained cells using 2% uranyl acetate of strains KJ51-3^T (A) and 15G1-11^T (B) cultivated on marine agar at 30°C for 2 days.

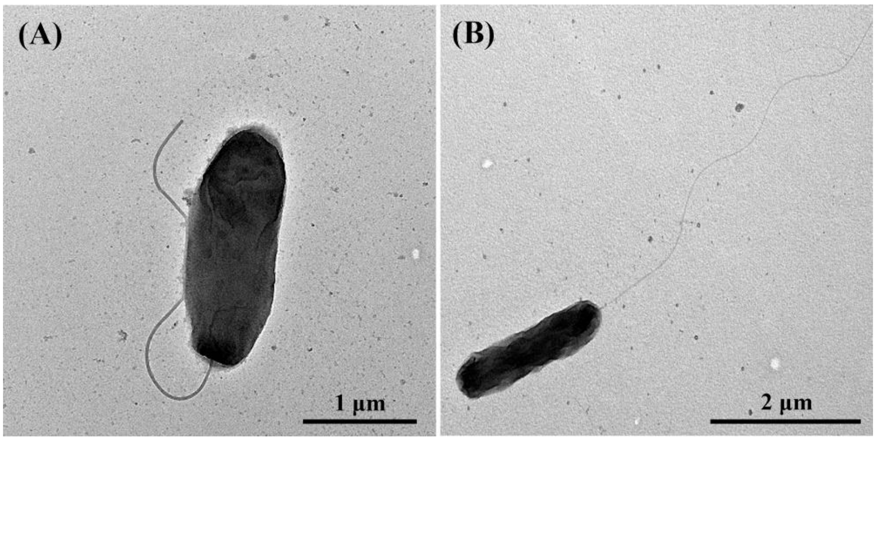

Fig. S3. Two-dimensional thin-layer chromatograms (TLC) showing the polar lipid profiles of strains KJ51-3^T and 15G1-11^T. Solvent systems: (I) chloroform-methanol-water (65:25:4, v/v/v) and (II) chloroform-acetic acid-methanol-water (80:15:12:4, v/v/v/v). The TLC plates were sprayed with 10% ethanolic molybdophosphoric acid (A), ninhydrin (B), Dittmer-Lester (C), and α-naphthol/sulfuric acid (D) reagents for the detection of total polar lipids, aminolipids, phospholipids, and glycolipids, respectively. DPG, diphosphatidylglycerol; PG, phosphatidylglycerol; PE, phosphatidylethanolamine; AL, unidentified aminolipid; PL, unidentified phospholipid.

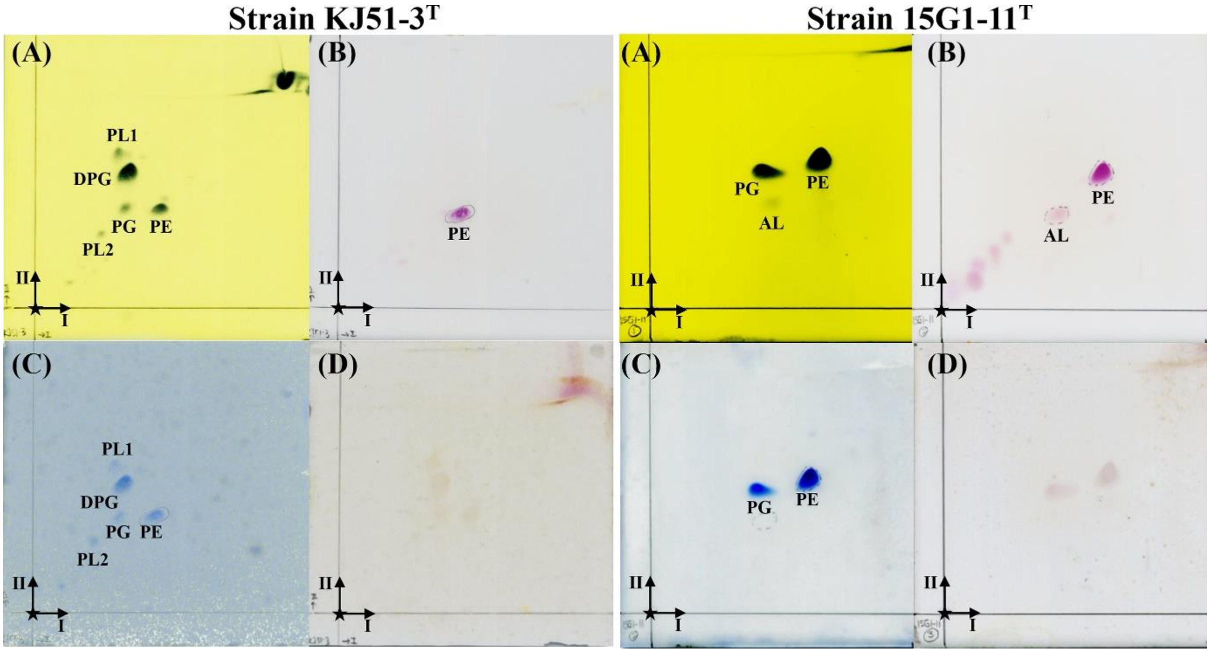

Table S1. Genome relatedness among strains KJ51-3^T and 15G1-11^T and their closely related *Marinomonas* type strains

Taxa: 1, strain KJ51-3^T (JAPEUL000000000); 2, strain 15G1-11^T (JAPUBN000000000); 3, *M. arctica* 328^T (QHJF00000000); 4, *M. algicola* SM1966^T (CP061941); 5, *M. communis* DSM 5604^T (SNZA00000000); 6, *M. shanghaiensis* DSL-35^T (QGOK00000000). The genomes of strains KJ51-3^T and 15G1-11^T were sequenced in this study.

		dDDH† value (%)					
		**1**	**2**	**3**	**4**	**5**	**6**
ANI† value (%)	**1**	–	21.5	46.6	21.2	19.6	28.8
	**2**	70.7	–	20.6	22.7	19.7	19.7
	**3**	91.9	70.8	–	20.6	18.8	30.9
	**4**	70.8	79.3	70.9	–	19.9	20.1
	**5**	70.6	69.3	70.7	69.2	–	18.6
	**6**	85.2	70.9	86.5	70.9	70.7	–

†ANI, average nucleotide identity; dDDH, digital DNA-DNA hybridization.

Table S2. Cellular fatty acid compositions (%) of strains KJ51-3^T and 15G1-11^T and closely related type strains of the genus *Marionomonas*

Taxa: 1, strain KJ51-3^T; 2, strain 15G1-11^T; 3, *M. arctica* 328^T; 4, *M. algicola* SM1966^T; 5, *M. communis* DSM 5604^T. All data presented herein were derived from this study. Data are expressed as percentages of the total fatty acids, and fatty acids constituting less than 1.0% in all strains are not shown. Major components (>5.0%) are highlighted in bold; symbols: tr, trace amount (<1.0%); –, not detected.

**Fatty acid**	**1**	**2**	**3**	**4**	**5**
Saturated:					
$C_{10:0}$	1.6	1.1	1.5	1.7	2.6
$C_{12:0}$	2.1	1.0	tr	3.9	**5.4**
$C_{14:0}$	1.0	tr	-	1.7	2.0
**$C_{16:0}$**	**14.1**	**10.1**	**12.1**	**13.3**	**8.0**
$C_{18:0}$	4.8	2.4	2.6	1.3	1.4
Hydroxy:					
**$C_{10:0}$ 3-OH**	**17.3**	**5.9**	**24.2**	**5.3**	**14.3**
$C_{12:0}$ 3-OH	1.8	tr	1.2	3.9	tr
$C_{12:1}$ 3-OH	tr	2.8	tr	2.2	-
Summed feature*:					
**3**	**12.0**	**34.2**	**19.4**	**27.1**	**23.9**
**8**	**37.6**	**40.8**	**33.7**	**48.5**	**46.9**

*Summed Features are fatty acids that cannot be resolved reliably from another fatty acid using the chromatographic conditions chosen. The MIDI system groups these fatty acids together as one feature with a single percentage of the total. Summed feature 3, $C_{16:1}$ $\omega 7c$ and/or $C_{16:1}$ $\omega 6c$; summed feature 8, $C_{18:1}$ $\omega 7c$ and/or $C_{18:1}$ $\omega 6c$.
